# Bidirectional Relationship between HIV/HBV Infection and Comorbid Depression and/or Anxiety: A Systematic Review on Shared Biological Mechanisms

**DOI:** 10.3390/jpm13121689

**Published:** 2023-12-05

**Authors:** Michele Fabrazzo, Salvatore Cipolla, Mariantonietta Pisaturo, Alessio Camerlengo, Paola Bucci, Pasquale Pezzella, Nicola Coppola, Silvana Galderisi

**Affiliations:** Department of Mental and Physical Health and Preventive Medicine, University of Campania “Luigi Vanvitelli”, 80138 Naples, Italy; salvatore.cipolla@studenti.unicampania.it (S.C.); mariantonietta.pisaturo@unicampania.it (M.P.); alessio.camerlengo@studenti.unicampania.it (A.C.); paola.bucci@unicampania.it (P.B.); pasquale.pezzella@unicampania.it (P.P.); nicola.coppola@unicampania.it (N.C.); silvana.galderisi@unicampania.it (S.G.)

**Keywords:** chronic inflammatory infectious diseases, HIV, HBV, shared biologic mechanisms, depression, anxiety

## Abstract

Background: Mental disorders that are comorbid with chronic infectious diseases may worsen clinical outcomes and patients’ quality of life. We hypothesized that depression and/or anxiety syndromes or symptoms comorbid with human immunodeficiency virus (HIV) or hepatitis B virus (HBV) infection might stem from shared biological mechanisms. Methods: We conducted a systematic review applying the PRISMA statement by searching into the PubMed, APA PsycInfo, and Scopus databases. We examined the literature on HIV/HBV infection comorbid with depression and/or anxiety in adults ≥18 years. Results: Thirty-one studies on HIV and three on HBV were analyzed. The Tat protein contributed to HIV-associated mood disorders due to the protein’s ability to cause neurodegeneration and induce hypothalamic–pituitary–adrenal (HPA) axis dysregulation in response to natural stressors. The decreased brain-derived neurotrophic factor (BDNF) levels also emerged as a mechanism involved in HIV neuropathogenesis and the associated mood symptoms. Neuroinflammation was implicated in depression and/or anxiety onset in patients with HIV/HBV infections. Microglial activation and release of cytokines, in particular, appeared as potential pathogenetic mechanisms. Furthermore, an altered balance between quinolinic acid and kynurenic acid production emerged in HIV patients with comorbid depression, indicating a glutamatergic dysfunction. Inflammatory cytokine production and the downregulation of cellular immune responses contributed to persisting inflammation, delayed healing, and functional decline in patients with chronic hepatitis B (CHB) infection. A shift in type 1–type 2 cytokine balance might be implicated in HBV-related immune pathogenesis, and depression and anxiety might be considered immunomodulatory factors. Cytokines also caused HPA axis hyperactivity, frequently observed in HIV/HBV patients with comorbid depression/anxiety. Conclusions: The present systematic review showed, for the first time, that HIV/HBV and depression and/or anxiety might have several biological mechanisms as common denominators. The longitudinal course of the highlighted biological mechanisms should be explored to establish the causative interrelationship among the involved mechanisms. In addition, future research should investigate the possibility that a patient’s clinical outcome might improve using pharmacological treatments acting on the biological mechanisms we described as common denominators of chronic inflammatory infective diseases and depression/anxiety.

## 1. Introduction

Human immunodeficiency virus (HIV) infection and acquired immune deficiency syndrome (AIDS) are significant global health burdens. According to the World Health Organization (WHO) [1], by the end of 2021, 38.4 million people worldwide lived with HIV, with 1.5 million new infection cases and 650,000 deaths. People living with HIV (PLWH) are forced to deal with an increased risk of mental symptoms/disorders, mainly anxiety and depression, possibly associated with perceived stigma and consequent social withdrawal, insomnia, and guilt feelings [2,3,4]. HIV patients face an alarming prevalent depression compared to the general population, with 30–50% presenting comorbid depressive symptoms [5]. The risk of depression in PLWH is estimated to be twice that of the general population [6], and suicide ideation is considerably high, consistent with the depression rates [3,4,7].

Hepatitis B infection is the most widespread infection in the WHO Western Pacific Region (116 million people are chronically infected), in the African Region (81 million), and in the Eastern Mediterranean Region (60 million). Hepatitis B virus (HBV) infection contracted in adulthood leads to chronic hepatitis in less than 5% of cases, whereas in infancy and early childhood it is in 95% of cases. In 2019, hepatitis B caused 820,000 deaths, primarily due to cirrhosis and hepatocellular carcinoma (WHO, 2017). Nikbakht Dastjerdi et al. showed that chronic hepatitis B (CHB) patients might suffer from depression (36.6%) and anxiety (40%) and experience a high psychopathological burden [8]. Furthermore, in CHB patients and HBsAg-positive inactive carriers, comorbid anxiety symptoms appear to be more severe than in the general population, differently from comorbid depressive symptoms, which are more serious solely in the case of HBsAg-positive inactive carriers [9].

Depression and anxiety increase the risk of contracting infectious diseases and reactivating latent infections. Tsai et al. revealed that patients with HBV infection affected by comorbid depression/anxiety had a 1.189-fold increased risk of hepatitis B flares compared to the control cohort throughout a 16-year follow-up, with a higher incidence in the first year [10]. Major depression impacts the clinical outcomes and quality of life (QoL) of people with HIV or HBV and might impede treatment adherence. Comorbid depression might contribute to the progression of HIV infection toward increased morbidity and mortality [11,12,13,14]. In addition, psychosocial and lifestyle factors might increase the burden associated with depression in PLWH [15,16,17].

In the general population, depression and/or anxiety severity are associated with acute and chronic systemic inflammation markers, which might promote a susceptibility to depression [18,19,20,21,22,23,24,25,26]. Psychological distress and the associated negative emotions, such as anxiety, may also impact the B- and T-cell-mediated immune system and increase the risk of contracting infectious diseases and/or reactivating latent viral infections [27,28]. Low-grade inflammation and cytokines hypersecretion in the peripheral blood are consistently associated with HIV infection and related comorbidities [29]. Therefore, a central issue is establishing whether peripheral markers reflect pathological mechanisms in the central nervous system (CNS) or neuroinflammation results from primary peripheral inflammatory mechanisms.

A few authors hypothesized that deficits in serotonergic or dopaminergic pathways might disclose a depression risk [30,31]. Growing evidence supports that HIV-associated depression might relate to the modulatory effects of immune responses leading to cytokine-mediated inflammatory processes and to the activation of predominant stress pathways, such as the hypothalamic–pituitary–adrenal (HPA) axis and the sympathetic nervous system (SNS), which stimulate immune cells both in the periphery and in the CNS [32]. The “cytokine hypothesis of depression” suggested that inflammatory cytokines might act as neuromodulators and mediate behavioral, neuroendocrine, and neurochemical features of depressive disorders [33,34,35,36]. Moreover, several studies concluded that nonsteroidal anti-inflammatory drugs (NSAIDs) had both anti-inflammatory and antidepressant effects by inhibiting proinflammatory cytokines [37]. Contrarily, other studies proved that NSAIDs administered to patients with comorbid inflammatory diseases and depression might cause severe adverse effects. Furthermore, the cotreatment of NSAIDs with SSRIs was proved to attenuate the effects of antidepressant drugs [38,39,40,41].

Chronic inflammation and depression emerged to be associated also with increased corticotropin-releasing hormone (CRH) and glucocorticoid receptor resistance [42]. Both processes, in turn, contribute to increased inflammatory cytokines and persistent inflammation [21].

Further evidence suggests that depression etiology might be related to neuroinflammation through a set of immune responses in the CNS, characterized by microglial activation and inflammatory cytokines release [43,44].

Depression and inflammation appeared to be associated, and HIV infection in the brain elicited a neuroinflammatory response, possibly contributing to causing frequent comorbid depression and HIV infection in PLWH [29].

Our review shows evidence that the hypothesized frequent comorbidity between depression, anxiety symptoms or syndromes, and HIV and HBV, is ascribable to a shared pathophysiology. We conducted a systematic review of the clinical- and population-based literature relevant to HIV and HBV infections comorbid with depression and/or anxiety in adults ≥18 years and hypothesized that they shared underlying biological mechanisms.

## 2. Materials and Methods

We complied with the Preferred Reporting Items for Systematic Review and Meta-Analysis (PRISMA) 2020 statement [45], as applicable, and examined studies including patients ≥18 years diagnosed with HIV/HBV-comorbid syndromes or symptoms of depression and/or anxiety. The study was not registered.

We aimed to answer the following research questions:

1. Which biological mechanisms might explain the comorbidity between the chronic infectious disorder (HIV or HBV) and mood disorders/symptoms in patients whose primary diagnosis was HIV or HBV infection?
2. What are the gaps in the literature regarding this topic?
3. What suggestions can we provide for future studies?

We searched the PubMed, APA PsycInfo, and Scopus databases for relevant trials on animals and humans, with English as a language filter. The date limits were set from inception to June 2023. The search terms (MeSH headings) included ((“Depression”[MeSH Terms] OR “Depressive Disorder”[MeSH Terms] OR “Affective Symptoms”[MeSH Terms] OR “Anxiety Disorders”[MeSH Terms] OR “Anxiety”[MeSH Terms]) AND (“HIV Infections”[MeSH Terms] OR “Hepatitis B”[MeSH Terms]) AND (“Biological Factors”[MeSH Terms] OR “Inflammation”[MeSH Terms] OR “Immune System Phenomena”[MeSH Terms] OR “Amino Acids, Peptides, and Proteins”[MeSH Terms]) AND (“loattrfull text”[Filter] AND “english”[Language])) AND ((fft[Filter]) AND (english[Filter])). In addition, we hand-searched the reference lists of the included articles to detect any further relevant studies.

### 2.1. Eligibility Criteria

As mental disorders, we selected depression and anxiety (disease and/or symptoms), assessed through standardized psychopathological rating scales, regardless of clear-cut anxiety or depressive disorder based on Diagnostic and Statistical Manual of Mental Disorders (DSM) criteria. We extracted all the original studies on comorbidity between HIV or HBV infection and/or depression/anxiety that reported the severity of such conditions through outcome measures (i.e., clinical evaluation and/or blood samples testing for HIV/HBV infections, depression and/or anxiety psychopathological assessment scales). We included the following: (a) randomized controlled trials (RCTs) and longitudinal, cross-sectional, case–control, and cohort studies in patients ≥ 18 years, of either gender, with a clinical or laboratory diagnosis of HIV or HBV infection concurrent with depression and/or anxiety, assessed with a psychopathological rating scale; (b) studies on patients treated with approved antiretroviral therapy (ART) for HIV infection or drugs used in HBV-infected patients; (c) studies including patients cotreated with antidepressants and followed up for at least 12 weeks, the usual interval for resolving long-lasting mood symptoms; and (d) basic research studies on the biological mechanisms of animals infected with artificially induced corresponding human HIV or HBV, analyzing, using appropriate tests, the resulting depression- and anxiety-like behaviors. Finally, we excluded studies that were not in English, did not include subjects with proven HIV or HBV infection, did not explore the biological mechanisms shared by HIV/HBV infection and psychiatric disorders such as anxiety and/or depression, concerned pregnant women, or subjects with other medical conditions that were confounding factor for our analysis. Furthermore, we excluded reviews, letters, and editorials.

### 2.2. Selection of Studies

The titles and abstracts of articles were initially screened for inclusion and the relevant data extracted and presented in a tabular format. Finally, data on study characteristics, outcome measures, and type of therapeutical intervention, when applicable, were classified. The search strategy and selection process were displayed as a flow diagram and text, while the selected studies’ description as text and tables. The main results regarding each research question were summarized, and the study limitations, literature gaps, and areas needing further investigation were highlighted.

During the selection process, some interventions may have influenced the results. In particular, the use of only three search engines and the use of very specific keywords, on the one hand, may have reduced the number of off-topic results, while, on the other hand, may have resulted in the exclusion of relevant studies.

### 2.3. Quality of the Studies and Risk of the Bias Assessment

Two authors (S.C. and A.C.) independently assessed the quality of the non-randomized studies of interventions (NRSIs) and the risk of bias, through the Risk of Bias in Non-randomized Studies of Interventions (ROBINS-I) [46]. The tool comprises three main domains for bias evaluation—pre-intervention, during the intervention, and post-intervention—assessing and classifying the risk of bias for each domain and subdomain as low, moderate, high, or no information (Supplementary Tables S1 and S2). The two authors resolved disagreements by discussing or involving a third author (M.F.). The authors considered an NRSI as follows: (a) as low risk (if at a low risk of bias for all the domains); (b) as moderate risk (if at a moderate risk for at least one domain); (c) as high risk (if at a high risk of bias for at least one domain but not at a critical risk of bias in any domain); and (d) as critical risk (if at critical risk in at least one domain). Also, “no information” was displayed for an NRSI when no exact evaluation of a high or critical risk of bias was achievable and/or information about key domains was missing.

## 3. Results

We initially extracted 806 articles, from which we selected 40 articles potentially relevant for full-text screening and excluded 6 that did not address biological mechanisms relevant to our research questions. Supplementary Figure S1 presents the flow chart of the selected studies and describes the reasons for the exclusion.

Among the remaining 34 studies, 31 presented results on shared biological mechanisms associated with HIV infection and depression and/or anxiety, and 3 focused on patients with HBV infection. Specifically, three preclinical studies on animal models reported shared biological mechanisms of HIV infection with depression-like or anxiety-like behaviors; six genetic studies analyzed genotype distribution and allele frequency in patients with comorbid HIV infection and depression or anxiety, and twenty-two non-randomized clinical reports evaluated common biological mechanisms in patients with comorbid HIV infection and depressive disorders (Table 1). The remaining three studies reported shared biological mechanisms involved in HBV-infected patients with comorbid anxiety or depression (Table 2).

The overall risk of bias was moderate for most of the included NRSIs in HIV patients—16 studies with moderate and 12 with high risk (Supplementary Table S1). Instead, the overall risk of bias for the NRSIs in HBV patients appeared high for two of the included studies and moderate for the other one (Supplementary Table S2).

### 3.1. Studies on HIV Infection

#### 3.1.1. Preclinical Studies

The preclinical studies focused on the effects of the transactivator of transcription (Tat) regulatory protein associated with HIV-1 infection that might contribute to HIV-associated mood disorders. In one of the studies, the Tat protein activated brain cytokine signaling and led to the increased expression of indoleamine 2,3-dioxygenase (IDO) in male mice, thus causing a depressive-like behavior [47] (Table 1). On the other hand, Tat protein and morphine might differently induce depressive-like behaviors associated with increased neuroinflammation, synaptic losses, and immune fatigue within the prefrontal cortex of male mice [49]. Furthermore, the enhanced expression of the Tat 1–86 protein also caused dose- and time-dependent anxiety-like effects in the (GT-tg) bigenic mice of study [48] (Table 1).

#### 3.1.2. Genetic Studies

A correlation between depression severity and FK506-binding protein 5 (FKBP5) gene expression (a negative regulator of the glucocorticoid receptor gene) in individuals homozygous for GG of the FKBP5 single nucleotide polymorphism (SNP) rs3800373 was emphasized [51]. Deficits in glucocorticoid signaling (decreased nuclear translocation of the glucocorticoid receptors and a lower transcription of FKBP5), instead, were synergistically associated in women with comorbid HIV and depressive symptoms [59] (Table 1). In addition, the authors highlighted an association between depressive symptoms and HIV serostatus in baseline and dexamethasone-stimulated conditions in patients with elevated baseline expression of FKBP5 and nuclear receptor subfamily 3 group C member 1 gene (Nr3c1). Furthermore, BDNF receptors were reported to be involved in the depression pathophysiology of patients with HIV infection. A particular SNP in the neurotrophic tyrosine kinase receptor type 2 (NTRK2 gene, encoding the receptor complex formed by TrkB) might contribute to comorbid HIV infection and major depressive disorder (MDD) in individuals with different ethnic backgrounds [54,67].

In HIV-infected patients, the comorbidity between neuroinflammation and psychopathological symptoms might be mediated by imbalances in the kynurenic pathway (KP). Two key KP enzymes are the kynurenine-aminotransferase II (KATII), which yields antioxidative kynurenine acid (KYN A) in astrocytes, and the kynurenine-3-monooxygenase (KMO), which produces neurotoxic metabolites in microglia. Polymorphisms of KYN pathway-related genes, such as the C-allele variant of KATII-rs1480544, appeared to be associated with psychopathological distress in HIV-positive patients, who consequently showed more severe depressive and anxiety symptoms and greater neuroinflammation [57]. Borghetti et al. found that HIV-positive CA genotype carriers of the allele variant of the SLC22A2 808 gene (C to A polymorphism), encoding the organic cation transporter 2 (OCT2), were at higher risk of manifesting anxiety and hostility [60]. OCT2 is a low-affinity carrier for various physiological compounds, including catecholamines, serotonin, and choline neurotransmitters, besides xenobiotics, as heavy metals, antiviral drugs, and beta-lactam antibiotics in mammals. Also, OCT2 takes part in the postsynaptic reuptake of the extraneuronal neurotransmitters, especially catecholamines, which escape from the high-affinity transporters. Moreover, OCT2 is essential in modulating the response to stress by inhibiting the corticosterone release driven by the HPA system, thus reducing stress and depression-like behaviors.

#### 3.1.3. Studies Investigating Chronic Inflammatory Mechanisms

Chronic, low-level CNS inflammation is a primary mechanism contributing to HIV neuropathogenesis. HIV enters the brain 4–8 days after peripheral infection and the infected cells produce chemokines, cytokines, neurotoxic mediators, and viral proteins that contribute to chronic inflammation and ongoing neuronal damage. Concerning such an issue, Praus et al. described a positive correlation between elevated levels of anxiety or depression and cerebrospinal fluid (CSF) cells and proteins [50]. Saloner et al. suggested that depression pathophysiology in HIV-positive patients was associated with low central dopaminergic activity and neuroinflammation [64]. On the other hand, Woods et al. concluded that the neurotoxic effects of the HIV protein gp120 are responsible for reducing the availability of BDNF in PLWH [68].

Neopterin, a catabolic product of the purine nucleotide guanosine triphosphate (GTP), synthesized by human macrophages upon stimulation with the cytokine interferon-gamma (IFN-γ), is indicative of a pro-inflammatory immune status. The measurement of neopterin concentrations in body fluids provides information about the activation of cellular immune responses in humans under the control of T helper type 1 (TH1) cells. A high neopterin production is associated with the increased synthesis of reactive oxygen products, which indirectly allows the assessment of the oxidative stress’ extent elicited by the immune system. An increased neopterin production may also correlate with MDD and subclinical depressive symptoms in acutely infected HIV patients. Warriner et al. reported that neopterin plasma levels might act as systemic immune markers useful in identifying treatment-resistant HIV-positive patients at greater risk of developing chronic depression [52] (Table 1). On the other hand, Saloner et al. highlighted that plasma levels of neopterin were associated with lifetime MDD but not with ongoing depressive symptoms in such patients [71]. The authors concluded that the association might be related to the amino acid metabolism and neurotransmitter synthesis disruptions occurring in the inflammatory process. Otherwise, Anderson et al. hypothesized a contribution of peripheral macrophage activation in comorbid depression pathogenesis in PLWH [28]. The authors reported that sCD163, a biomarker of macrophage activation, was higher in the plasma of HIV-positive patients and significantly associated with severe depressive symptoms compared to HIV-negative patients (Table 1).

Furthermore, HIV-positive patients might also present high degrees of psychological distress. In this regard, Fumaz et al. reported that high plasma levels of IL-6 were closely associated with psychological stress and anxiety/depression symptoms [53]. In addition, Petersen et al. highlighted a correlation between IL-6 and depression exclusively in men, suggesting that the depression–inflammation link differed based on sex [74]. Bekhbat et al., instead, noticed that depressive symptoms were independently associated with the reduced lipopolysaccharide (LPS)-induced release of IL-6 and TNF-α, in addition to the elevated expression of FKBP5 and Nr3c1 genes [59].

The C-reactive protein (CRP) has long been identified as an inflammatory marker associated with HIV. A linear relationship between serum CRP concentration and depressive symptoms severity appeared in HIV-positive patients. In particular, HIV-positive male patients with a high level of inflammation (CRP > 3 mg/L) showed a greater risk for depression [55]. In a sample of HIV-positive Tanzanian adults, Memiah et al. found that elevated levels of CRP and sTNFR-II were positively associated with self-reported mental health symptoms [66]. Moreover, Lu et al. suggested that immune activation might be involved in depression risk in both HIV-positive and HIV-negative male patients and that several markers might be associated with higher odds of depressive symptoms (Table 1) [61].

Overall, a recent study hypothesized that the association between HIV status and depressive symptoms might partly be attributable to central and peripheral inflammatory biomarkers [75]. Specifically, these biomarkers are the monokine induced by interferon (MIG) and the TNF-α in the plasma and the macrophage inflammatory protein-1 alpha (MIP1-α) and the IL-6 in the CSF, all of which proved to be potential mediators of such an association. Rubin et al. suggested that both hormonal and inflammatory mechanisms might be implicated in cognitive impairments in patients with HIV presenting MDD or remitted MDD (rMDD) [63]. In particular, in HIV female patients, basal cortisol concentrations were higher in rMDD versus no-MDD groups, while basal inflammatory cytokines were higher in rMDD versus no-MDD male and female patients with HIV (Table 1). Based on their findings, Derry et al. suggested potential links among depression, inflammation, and health in elderly HIV-positive patients who showed higher serum levels of cytokines and more depressive symptoms, along with a worse physical function, when compared to HIV-positive patients with fewer depressive symptoms and lower cytokine plasma levels [70]. Differently, Zuñiga et al. reported that, in HIV-positive patients, the most relevant depression biomarker was the increased plasma levels of TNF-α [65]. Furthermore, Musinguzi et al. noticed that IL-6 and TNF-α, rather than CRP, were significantly and positively associated with MDD [58]. According to Saylor et al., serum IL-6 appeared to be highly associated with advanced immunosuppression, depression, and HIV-associated neurocognitive disorder in a cohort of HIV-positive patients [62]. Instead, Yang et al. suggested a role for endogenous cannabinoid signaling in the modulation of depression in HIV-positive patients [72]. A down-regulation of cannabinoid receptor 1 (CBR1), associated with an increased expression of nucleobindin 1 (NUCB1) protein in the temporal cortical neurons, might be responsible for the comorbid mood disorder in the above-mentioned study.

Finally, Taylor et al. suggested that gut microbiome changes and gut mucosal barrier dysfunctions might expose PLWH to proinflammatory microbial products, which might act in neuroinflammation, and increase the risk of MDD [76].

#### 3.1.4. Studies Investigating Monoamine Metabolites and Hormones

Depressive symptoms have been associated with augmented IFN responses and increased tryptophan catabolism in HIV patients. Such findings suggested that minor alterations in the metabolism of monoamines and mitochondrial energetics might contribute to the activation of mechanisms that favor MDD’s onset. Such mechanisms, in turn, are possibly influenced by inflammation during HIV infection [56]. Drivsholm et al. reported that an altered balance between quinolinic acid (QA) and KYN A production was associated with depression in HIV patients [69]. Tryptophan (Trp) is metabolized into KYN through IDO-1, whose activity is enhanced in HIV patients. KYN, in turn, is metabolized via kynurenine aminotransferase (KAT) and KMO, resulting in either KYN A or 3-hydroxykynurenine and QA. Indeed, reduced KYN and KYN A and an increased QA and QA/KYN A ratio are also the most consistently reported findings in patients suffering from depression without infection. In addition, KYN A might act as an antagonist and QA as an agonist of N-methyl-D-aspartate (NMDA) receptors. Altered concentrations of KYN metabolites may lead to glutamatergic dysfunction and, consequently, depression. Such findings suggested that the increased activity of KMO contributed to depression pathogenesis. Accordingly, Drivsholm et al. hypothesized that alterations in the KYN pathway might partly mediate a close relationship between macrophage-driven inflammation and depression in HIV infection [69]. Finally, Shortell et al. report a correlation between neuropeptide oxytocin and depressive symptoms in PLWH (Table 1) [73].

### 3.2. Studies on HBV Infection

Increasing evidence sustains that chronic psychophysical stress might suppress protective immune responses and/or exacerbate pathological immune responses by altering the type 1–type 2 cytokine balance, inducing low-grade chronic inflammation, and suppressing the numbers, trafficking, and function of immunoprotective cells [80].

Generally, type 1 cytokines (IL-2, IFN-γ, IL-12, and TNF-ß) favor the development of a strong cellular immune response, whereas type 2 cytokines (IL-4, IL-5, IL-6, IL-10, and IL-13) a strong humoral immune response. A few type 1 and type 2 cytokines are cross-regulatory. For example, IFN- γ and IL-12 might decrease type 2 cytokine levels, whereas IL-4 and IL-10 might decrease type 1 cytokine levels [81]. Indeed, in patients with CHB, He et al. detailed a marked increase in the plasma levels of IL-10 associated with a decrease in the IFN-γ plasma levels, due to a shift in the type 1–type 2 cytokine balance toward a type-2 response [77]. The authors concluded that, in patients with HBV infection, chronic stress and associated anxiety might contribute to the shift, which possibly played a role in the HBV-related immune pathogenesis (Table 2). The study of Bahramabadi et al. highlighted that chronic inflammation might be related to the downregulation of transforming growth factor (TGF)-β in patients with CHB [78]. Moreover, the results confirmed the high prevalence of depression and its role in the downregulation of TGF-β in CHB patients. Accordingly, depression might lead to reduced TGF-β plasma levels and, subsequently, to inflammation in CHB patients through the decreased serum levels of IL-6 and TGF-β. Finally, Safari-Arababadi et al. evaluated the relationship between depression and anxiety with the expression of some inflammatory transcription factors, which are the receptor-interacting protein 1 (RIP1) and the IFN-β promoter stimulator-1 (IPS-1) in the peripheral blood mononuclear cells of CHB patients, known as the critical molecules against HBV [79]. The authors hypothesized that depression might be associated with an altered expression of IPS-1 sex-dependently and that only IPS-1 significantly decreased in the male CHB patients with depression compared to the patients without depression (Table 2).

## 4. Discussion

The present systematic review aimed to analyze the possible pathophysiological mechanisms underlying comorbid HIV/HBV infections and mental syndromes or symptoms of depression and anxiety to identify the potential literature gaps. For the first time, we recognized several biological mechanisms as common denominators of the hypothesized reciprocal/bidirectional relationship between HIV/HBV and depression/anxiety. However, it remains unclear whether such mechanisms are the expression of peripheral inflammatory processes, with the subsequent involvement of microglia activation, decreased neurogenesis, and increased apoptosis, or rather primary CNS processes extending to peripheral organs. Most studies lack the longitudinal course and the causal relationship with the comorbid processes.

The literature primarily focused on comorbid mental disorders in HIV patients, rather than HBV infection. A few reviews have reported on inflammatory mechanisms as a possible link between depression/anxiety and chronic infectious diseases [82,83,84]. In particular, HIV might predispose infected individuals to depression via interrelated mechanisms, and neuroinflammation might contribute to the highly prevalent mood disorders in PLWH. Further reviews, moreover, revealed that, in HIV patients with concurrent psychopathological symptoms, ART possibly attenuated virus-associated neuroinflammatory activities and acted on the shared biological mechanisms of the comorbid conditions [85].

The literature analysis highlighted that HIV-1 replication in the CNS initiated after invading monocytes and CD4+ T cells and that the virus spread to microglial cells and astrocytes within the brain parenchyma [86]. In addition, infected macrophages entered the brain in low abundance during early HIV-1 infection and activated microglial cells to initiate producing and releasing cytokines [32]. Furthermore, overactivated microglial cells were involved in exacerbating neuronal injury through the synthesis and secretion of proinflammatory and cytotoxic factors [87]. Also, the literature shows that several HIV proteins might interact with neurons, microglia, and astrocytes, further contributing to neuroinflammation. HIV proteins, including Tat gp120, Vpr, and Nef, might directly induce neuronal damage [88,89,90,91], while activated microglia might contribute to neurodegeneration by releasing cytokines and toxins, which damage neurons and astrocytes [92,93].

Preclinical studies suggest that the activation of the Tat regulatory protein might be involved in the underlying mechanisms of depression- and anxiety-like behaviors in mice models of HIV infection [47,48,49] and lead to dendritic pruning, decreased spine density, and synapse loss [94]. Tat mRNA and proteins were also found in the CNS of patients with HIV-associated neurocognitive disorders [95]. Furthermore, the extent of cognitive decline might correlate with dendritic damage and synapse loss rather than with neuronal death [96]. The functional Tat protein promotes cytokine production and infiltration, oxidative stress, and neurotoxicity [32]. Also, the Tat protein might contribute to dysregulating the HPA system in response to natural stressors, thus producing significantly high corticosterone levels and potentiating psychomotor and anxiety-like behaviors [97].

BDNF, a neurotrophic factor whose receptors are crucial in depression pathophysiology, appeared to be implicated in mood symptoms observed in HIV disease. Indeed, positive PLWH showed significantly lower BDNF plasma levels when compared with negative PLWH [68] due to the reduced processing of proBDNF into mature BDNF [54]. In addition, genetic factors, such as rs6265 polymorphism in the BDNF gene, might contribute to lower BDNF levels in HIV-1-positive individuals. Furthermore, Avdoshina et al. reported that a particular SNP in the NTRK2 gene might contribute to HIV infection and major depressive disorder (MDD) comorbidity in individuals with different ethnic backgrounds [54].

One primary mechanism contributing to HIV neuropathogenesis is the chronic inflammation in the CNS, with HIV-infected microglia responding vigorously to proinflammatory signals and producing excessive cytokines [86]. Elevated proinflammatory cytokines, such as TNF-α, IL-6, and IL-1β, appeared in the plasma and lymph nodes from the early stage of HIV-1 infection [82,98]. Indeed, several studies reported immune activation and increased plasma cytokines (IL-1 and IL-6) in the blood of HIV patients with depressive symptoms [29].

In light of the above considerations, chronic inflammation might contribute to depression pathogenesis also in PLWH.

The central action of inflammatory cytokines might also explain the HPA axis hyperactivity that is frequent in depressive disorders, which interferes with the inhibition of the negative feedback of circulating corticosteroids on the HPA axis. Furthermore, cytokines might reduce the 5-HT levels and lower tryptophan availability by activating the tryptophan-metabolizing enzyme IDO 2,3. The major effects of inflammatory cytokines might explain most symptoms in depressed patients, though it remains unclear whether cytokines are causative of depression or represent an epiphenomenon.

Under physiological conditions, cytokines, especially TNF-α and IL-1, provide essential trophic support to neurons, as they enhance neurogenesis, promote long-term potentiation, and maintain normal cognitive functions [32]. On the other hand, an excessive and/or prolonged activation of cytokine networks might cause diminished neurotrophic support, decreased neurogenesis, increased glutamatergic activation, oxidative stress, induction of apoptosis in relevant cell types (e.g., astrocytes and oligodendrocytes), and dysregulation of glial/neuronal interactions [32,85]. A few studies also identified imbalances in TNF-α, IL-1, and IL-6 as being implicated in mood and cognitive impairments [32,63].

The persistent CNS’ cytokine response might, therefore, seem essential in chronic immune activation in the CNS during HIV infection. Moreover, the levels of cytokines correlate with the progression of HIV infection and provide a basis for neurocognitive alterations [83]. Growing evidence has also confirmed the association between chronic inflammation and depression, manifested with increased C-reactive protein levels and proinflammatory cytokines, such as IL-1 β, IL-6, and TNF-α [18]. In particular, TNF-α levels appeared elevated in the serum and CSF of HIV patients [29]. Nevertheless, HIV infection itself might increase TNF-α levels, which, in turn, might impact viral replication [99]. Moreover, TNF-α proved crucial in activating the cytokine cascade in several inflammatory diseases and regulating the biological function of macrophages [100]. Also, IL-1β appeared decisive in the neuroinflammatory process associated with HIV-1 infection and was induced in human monocytes with subsequent inflammasome activation [101]. More precisely, IL-1β was induced in mononuclear phagocytes with the HIV envelope protein gp120 and, in turn, might induce HIV replication in the infected cells [85]. In addition, IL-1β appeared elevated in the plasma of HIV-infected patients, had neurotoxic effects on astrocytes and endothelial cells, and increased free radical and metalloproteinase production, which might cause neuronal death [29,102].

On the other hand, depression is associated with high levels of IL-1β, possibly responsible for activating a common pathway leading to depression and cognitive decline [83].

Based on our analysis, IL-6 might also present as a general biomarker of inflammation in HIV subjects, being implicated as a driver of ongoing CNS inflammation in virologically suppressed ART-treated patients. In addition, IL-6 might activate inflammatory cytokine expression in astrocytes and proved to be associated with depressive symptoms [52,53,62].

Chemokines were also responsible for neurological damage and inflammation, possibly contributing to depression onset [32]. Moreover, chemokines might act as ligands for HIV co-receptors and mediators of inflammatory responses, contributing to HIV neuropathogenesis by recruiting HIV-infected immune cells to the brain, thus facilitating viral entry into the cells [22,32].

We identified other mechanisms as potential common denominators between HIV infection and mood disorders. For example, reduced monoamines proved to be associated with depressive symptoms, including loss of motivation and energy, which decreased hedonic drive [32]. HIV was demonstrated to interfere with monoaminergic function via several mechanisms and act similarly with a few drugs of abuse. Indeed, HIV proteins might bind to the dopamine transporter (DAT) to block the neurotransmitter reuptake, thereby elevating dopamine levels and successively impairing the function of DAT. The accumulated synaptic dopamine levels, by binding to dopamine receptors, might activate adjacent microglia, possibly resulting in increased HIV replication and increased production of inflammatory mediators such as TNF-α and chemokines. In turn, increased HIV replication might lead to increased brain viral load and shedding of HIV proteins, gp120, and Tat. Such proteins, as well as TNF-α, might induce the cell death of adjacent dopaminergic neurons via apoptosis. The autoxidation and metabolism of accumulated synaptic dopamine might lead to the generation of reactive oxygen species (hydrogen peroxide), quinones, and semiquinones, which might also induce the apoptosis of neurons. Increased cell death of dopaminergic neurons might finally lead to a dopamine deficit, which potentially exacerbates the severity and/or accelerates the progression of HIV-associated mood and neurocognitive disorders [32].

Cytokinergic and monoaminergic mechanisms might also synergize when reducing serotonin levels during HIV infection. For example, TNF-α, IL-1β, and IL-6, as well as Tat and gp120, might activate the KYN pathway by stimulating glial IDO. Such an enzyme redirects the available dietary tryptophan to produce KYN, which, in astrocytes, is converted into KYN A and then transformed by activated microglia into 3-hydroxykynurenine (3HK) and QA. In addition, 3HK and QA might act as NMDA receptor agonists and directly contribute to excitotoxicity and Ca2+-mediated cell death. Differently, KYN A might act as an NMDA receptor antagonist, thus inhibiting glutamate release and providing a neuroprotective activity [32,85]. Such imbalanced metabolic processes in hyperactivated microglia might cause QA overproduction and consequent glutamate release. The metabolic processes, in turn, might lead to oxidative stress, mitochondrial dysfunction, and death of neurons, astrocytes, and oligodendrocytes [32]. Thus, QA might represent a highly excitotoxic marker of HIV-associated neurological diseases whose levels might reflect the extent of immune activation in blood and CNS [32].

Altered glutamatergic functions appeared to also be involved in depression pathogenesis [69]. Thus, altered concentrations of the KYN metabolites (3HK and QA) might lead to glutamatergic dysfunction and, consequently, to depression. A few studies analyzed the association between changes in the KYN pathway of tryptophan metabolism and HIV infection, though the modified KYN metabolism appeared to be associated with depression also in non-infected patients [103]. Drivsholm et al. reported higher levels of QA and QA/KYN A ratio in PLWH with depression when compared to PLWH without depression, suggesting that increased KMO activity might participate in depression pathogenesis [69]. In CNS, KMO was mainly expressed in the macrophages of the microglia, which is why Drivsholm et al. hypothesized that a relationship between macrophage-driven inflammation and depression in HIV infection might be partly mediated by the altered KYN pathway of tryptophan metabolism [69].

Our review also examined chronic HBV infection (CHB), a further example of the bidirectional association between inflammation and mood disorders.

The pathogenesis of CHB infection proved to be immune-mediated [84]. Innate and adaptive immune cells are crucial in controlling HBV infection, despite also appearing responsible for the inflammatory process. During the initial phase of HBV infection, innate immunity is activated and causes the production of antiviral cytokines. Such a process is followed by the activation and intrahepatic recruitment of the adaptive immune system, resulting in the elimination of the virus. In CHB infection, further alterations occur in innate and adaptive immunity, including increased regulatory cells, overexpression of coinhibitory receptors, and release of inflammatory mediators. The joint mechanisms prevail over the antiviral response, thus causing persistent viral infection and subsequent immune alterations associated with progression to fibrosis, cirrhosis, and hepatocellular carcinoma. In this regard, cytokines act as both inflammatory and anti-inflammatory molecules possibly contributing to the acute or chronic evolution of inflammatory conditions, whose persistence might trigger damaging and destructive immune responses leading to chronic medical conditions [104].

On the other hand, the continuous remodeling of the immune system throughout people’s life appeared to rely on the constant interaction between the cytokines’ network and a few factors (epigenetic, environmental, and lifestyle) [105]. He et al. observed in CHB patients markedly increased plasma levels of IL-10, a type 2 cytokine crucial to the pathogenesis of many fibrotic diseases, and decreased plasma levels of IFN-ɣ, a type 1 cytokine favoring the development of a strong cellular immune response [77]. The authors concluded that stress might be responsible for shifting the type 1–type 2 cytokine balance toward a type-2 response. Such a shift might implicate a role of psychological distress in HBV-related immune pathogenesis. A mechanism by which immune response against HBV is suppressed was reported to stem from the releasing of TGF-β, an anti-inflammatory cytokine produced during tissue remodeling and significantly increased in CHB patients compared to healthy controls [106]. TGF-β, associated with IL-6, participated in developing Th17, the primary cell type involved in inducing inflammation. Bahramabadi et al. suggested that chronic inflammation in CHB patients might relate to downregulated TGF-β and noticed that a highly prevalent depression might contribute to a decreased TGF-β [78]. Reciprocally, depression might lead to reduced TGF-β plasma levels and, subsequently, induce inflammation in CHB patients through decreased serum levels of IL-6 and TGF-β.

As reported in a few studies, IL-6, IL-8, and TNF-α are primary innate immune cytokines that induce inflammation and immune response against viruses [107]. Bahramabadi et al. reported that CHB patients might suffer from depression (33.4%) and anxiety (76.7%) and, compared with healthy controls, showed increased serum levels of IL-8, associated with decreased IL-6 and TGF-β levels [78]. Accordingly, they concluded that chronic inflammation in CHB patients might relate to downregulated TGF-β, through which depression might also induce inflammation. Therefore, depression and anxiety might be considered immunomodulatory factors. Also, Safari-Arababadi et al. confirmed a link between the host and the virus in depression and anxiety manifestation, which might alter immune-related molecule expression [79]. The authors showed that IPS-1 significantly decreased in male CHB patients with mild, moderate, or severe depression compared to patients without depression. However, the manifestation of some inflammatory transcription factors, such as IPS-1 and RIP1, did not change with severe depression and anxiety. Therefore, depression might be associated with the downregulation of IPS-1 in CHB patients depending on their gender. The results indicated depression as a potential factor participating in immune system alterations in male subjects. Furthermore, the hormonal differences between males and females and the effects of sex hormones on immune cell functions might lead to the hypothesis that such hormones participate in inducing various immune responses, which are still partly unknown [108].

## 5. Conclusions

The present systematic review showed, for the first time, that HIV/HBV and depression and/or anxiety might have several biological mechanisms as common denominators. In particular, the ability of the Tat regulatory protein to promote neurodegeneration, as well as decreased BDNF levels, microglial activation, and the release of inflammatory cytokines emerged as common potential pathogenetic mechanisms involved in the mood symptoms of patients with HIV infection.

A shift in the type 1–type 2 cytokine balance might be implicated in HBV-related immune pathogenesis, and depression, besides anxiety, might be considered as an immunomodulatory factor. In particular, depression might lead to reduced TGF-β plasma levels and subsequently induce inflammation through increased serum levels of IL-8, associated with decreased IL-6 and TGF-β levels. Additionally, depression might also be associated with the downregulation of IPS-1 in CHB male patients.

The longitudinal course of the highlighted biological mechanisms should be explored to establish the causative interrelationship among the involved mechanisms. For example, it remains unclear how peripheral mechanisms might involve central brain mechanisms and vice versa and, also, which biological factors might serve as a linking system to trigger macrophages and microglia activation.

Mental disorders associated with chronic inflammatory infective diseases might impair the response to treatment and further worsen the physical disease in question. Therefore, an early diagnosis of mental disorders in individuals with primary chronic infective diseases is decisive in order to administer specific treatments, enhance the patient’s general condition, and reduce health system costs.

Most studies we examined focused on the patient’s plasma levels of inflammatory markers of subclinical damage and the psychopathological status, assessed through self- and clinician-rated instruments.

Future research should evaluate the cognitive functioning of patients, the perception of stigma, and coping strategies, besides QoL, to determine clinical outcome improvement based on pharmacotherapies acting on the biological mechanisms shared by chronic inflammatory infective diseases and depression/anxiety.

## Figures and Tables

**Table 1:** Preclinical and clinical studies investigating shared biological mechanisms in patients with HIV and depression/anxiety symptoms/syndromes.

Authors, Year of Publication, Country of Study	Type of Study, Target Population	Infectious Induction/ Assessment	Psychiatric Assessment	Evaluated Biological Indices	Results
Lawson et al. 2011 USA [47]	Preclinical study of 16 three-month old male Balb/c mice vs. 58 three-month old male C57BL/6J mice	Mice were administered Tat-HIV transactivator of transcription via ICV injection.	Modified version of the forced swim test and sucrose preference test.	Gene expression: TNF, IL-1b, IL-6, IDO, CD11b, Iba1, MHCII, GFAP, GAPDH	A single ICV injection of Tat increased brain expression of IL-1β, TNF-α, IL-6, and IDO, causing a depressive-like behavior, but not a sickness behavior.
Paris et al. 2014 USA [48]	Preclinical study of 1032 Male GT-tg mice vs. 529 C57BL/6J as congenic, negative controls	GT-tg mice and C57BL/6J controls were administered doxycycline in a dose- or duration-dependent manner to induce Tat 1–86 in the brain.	Mice were assessed for anxiety-like behavior in an open field, social interaction, or marble burying task.	Western blot analysis of central expression of Tat 1–86 protein	Doxycycline significantly increased anxiety-like behavior in all tasks. Exposure to doxycycline higher doses impaired locomotor behavior.
Nass et al. 2023 USA [49]	Preclinical trial of79 adult male doxycycline-inducible HIV-1IIIB Tat 1–86 transgenic mice (3–5 months old) vs. Tat- control mice	Male mice treated with doxycycline for eight weeks to induce HIV-1 Tat expression; escalating doses of morphine or saline given only during the last two weeks.	Behavioral tests: Two-bottle choice; Sucrose preference; Novelty-induced sucrose hypophagia; Burrowing test; Nesting shredding/building test; Forced swim test; Novelty-suppressed feeding.	PFC chemokine/cytokine concentrations (CCL2, CCL3, CCL4, CCL5, CCL11, CXCL1, G-CSF, GM-CSF, TNFα, IFN-γ, IL-2, IL-3, IL-6, IL-9, IL-12p40, IL-12p70, IL-17A, IL-4, IL-5, IL-10, IL-13); spine density assessment	Tat, but not morphine, induced increased levels of the proinflammatory chemokine CCL3 in the PFC and decreased dendritic spine density on pyramidal neurons within layer V of the anterior cingulate cortex. Morphine expanded the Tat-induced increases in IL-10 within the PFC; Tat also tended to decrease the IL-6 and IFN-γ levels.
Praus et al. 1990 USA [50]	Longitudinal cohort study of98 HIV+ US Air Force members: 89 M, 9 F, mean age 28,9 years	Routine medical evaluation for HIV seropositivity.	HAM-DHAM-A	CSF nucleated cell count, total protein, and IgG level.Assessment of exposure to other neuropathic viruses (Epstein–Barr virus and cytomegalovirus).	In HIV+ patients, high levels of anxiety or depression positively correlated with CSF cells and proteins (nucleated cell count, total protein, and IgG level).
Tatro et al. 2010 USA [51]	Longitudinal cohort study CHARTER study of 54 HIV+ patients (40 M, 14 F) with MDD	Clinical evaluation and blood sample testing, either when an MDD episode was diagnosed or when the mood was euthymic.	BDI, CIDI, DSM-IV criteria for current and lifetime psychiatric diagnosis. Subjects longitudinally assessed for depression-remission patterns subgrouped as follows: Cohort 1 (*n* = 56): MDD at first visit, then euthymic at second visit. Cohort 2 (*n* = 30): euthymic at first visit, then depressed at second.	FKBP5 gene expression FKBP4 gene expression	A correlation between the severity of a depressive mood and FKBP5 gene expression emerged in individuals homozygous for GG of the FKBP5 SNP rs3800373.
Warriner et al. 2010 Canada [52]	Longitudinal cohort study of31 HIV+ ART-naïve patients: 26 M and 5 F, mean age 35.6 years	Clinical evaluation and laboratory assessment	PAOF Total; BDI; PFS-R; Neuropsychological test battery to assess attention/working memory, psychomotor efficiency, processing speed, and learning efficiency.	Serum concentrations of IL-6, TNF-α, neopterin. IL-6 mRNA expression.	Elevated neopterin plasma levels correlated with severe depressive symptoms in HIV+ patients. A subsample of antidepressant-treated patients continued to show severe depressive symptoms associated with elevated neopterin levels, thus representing treatment-resistant individuals.
Fumaz et al. 2012 Spain [53]	Cross-sectional study of 50 HIV+ individuals: 44 M, 6 F, mean age 39.0 years	Clinical evaluation and laboratory assessment	PSS-10 HADS	CD4 and CD8 cell count, CD4/CD8 ratio, and HIV RNA viral load IL-6 serum levels.	Individuals with psychological stress presented high levels of IL-6; psychological stress was the only variable remaining strongly associated with IL-6.
Avdoshina et al. 2013 USA [54]	Longitudinal cohort study of321 HIV− and 1109 HIV+ African Americans (non-Hispanic) and 50 HIV− and 256 HIV+ Caucasians (non-Hispanic), with a mean age of 50.6 years for the Caucasians patients and 50 years for the African Americans patients, enrolled at six consortium sites nationally.	Not reported	CES-D: score ≥ 16 used to characterize subjects as suffering from depression.	Genomic DNA analysis for allele distributions of rs1212171 (TRKB), rs2072446 (p75NTR), and rs56164415 (BDNF) polymorphisms.	Absence of depressive symptoms in HIV+ women associated with a genetic variation of TRKB (a tyrosine kinase receptor encoded by NTRK2) but not with the BDNF or p75NTR genes.
Poudel-Tandukar et al. 2014 Nepal [55]	Cross-sectional study of 316 HIV+ patients: 181 M and 135 F, mean age 33.9 years	Clinical assessment and blood tests	BDI-I using a symptom score ≥ 20, consistent with moderate to severe depressive symptoms requiring mental health intervention.	serum CRP	Linear relationship between serum CRP concentration and depression symptoms scores in HIV+ patients: HIV+ men with a high level of inflammation (CRP > 3 mg/L) showed a greater risk for depression.
Cassol et al. 2015 USA [56]	Cross-sectional study of 68 HIV+ vs. 36 HIV− patients, with a median age of 47 years, subgrouped as follows: HIV+ test cohort (*n* = 32); HIV+ validation cohort (*n* = 36); HIV− cohort (*n* = 36).	Not reported	BDI CES-D	Quantification of soluble markers in the plasma: IFN-α, IFN-γ, CXCL8, CXCL9, CXCL10, IL-1β, IL-6, IL-10, IL-12, TNF-α and CCL2.	Correlation of depressive symptoms’ severity with the plasma levels of decreased monoamine metabolites (phenylacetate, 4-hydroxyphenylacetate) and acylcarnitines (propionylcarnitine, isobutyrylcarnitine, isovalerylcarnitine, 2-methylbutyrylcarnitine) in both depressed HIV+ and HIV− patients. The depressive symptoms were associated with augmented IFN responses and increased tryptophan catabolism in HIV patients.
Douet et al. 2016 USA [57]	Cross-sectional study of 72 HIV− vs. 72 HIV+ patients	Confirmed HIV serostatus with documentation from medical records for HIV+, or a negative clear view HIV test for the HIV− participants	CES-D: score ≥16 used to identify those at risk for clinical depression; SCL90-R total and subitem scores, including the three main global indices (GSI, PST, and PSDI).	Genotyping assays for the KATII SNPrs1480544 and KMO SNPrs1053230. CSF KYNA: concentration determined only for 100 participants (51 HIV− and 49 HIV+)	The C-allele in KATII-rs1480544 appeared to be protective against psychopathological distress in HIV− but not in HIV+ patients, who had more psychopathological symptoms and greater neuroinflammation.
Musinguzi et al. 2018 Uganda [58]	Cross-sectional study of 201 patients, 41 M and 160 F, with a median age from 18 to >50 years, 62 (30.8%) with MDD	Medical records	MINI-Plus	IL-6, TNF-α, CRP	IL-6 and TNF-α serum levels were significantly and positively associated with MDD. Non-linear association between MDD and TNF-α serum levels at TNF-α concentrations above 500 pg/mL; a reverse relationship at TNF-α concentrations below 500 pg/mL.
Bekhbat et al. 2018 USA [59]	Cross-sectional study of147 African American F, <45 years old, subgrouped as follows: (1) HIV−, non-depressed (*n* = 37) and depressed (*n* = 34); (2) HIV+, non-depressed (*n* = 38) and depressed (*n* = 38).	Clinical interviews, physical examinations, laboratory testing every six months	CES-D: score ≥ 16, indicating a clinically relevant depressive symptom burden.	Glucocorticoid receptor expression (GR, gene: Nr3c1) and its negative regulator FKBP5 via quantitative RT-PCR from peripheral blood mononuclear cells (PBMCs) in baseline and dexamethasone-stimulated conditions.	Depressive symptoms and HIV serostatus independently associated with elevated baseline expression of FKBP5 and Nr3c1 and with reduced LPS-induced release of IL-6 and TNF-α.
Borghetti et al. 2019 Italy [60]	Cross-sectional study of203 HIV+ patients treated with a dolutegravir-containing regimen: 145 M and 58 F, mean age 51 years	Follow-up visit and blood sampling, plus a battery of tests to investigate neuropsychiatric symptoms.	SCL90-R (focusing on GSI) MINI-Plus subscale ad hoc self-reported questionnaire investigating symptoms occurring during the previous 4 weeks.	SLC22A2 808 C to A polymorphism. Dolutegravir plasma concentrations.	Prevalence of SLC22A2 CA genotype vs. CC genotype: in patients showing an abnormal GSI score (32.3% vs. 14.6%), anxiety (35.5% vs. 15.8%), hostility (32.3% vs. 16.4%), and moderate to severe headache (16.7% vs. 5.3%). Higher median dolutegravir concentration in patients with hostility (2019 vs. 1344 ng/mL) and psychoticism (2138 vs. 1383 ng/mL). No association of SLC22A2 genotypes and dolutegravir concentration with other neuropsychiatric dimensions, including suicide attempts or suicide ideation.
Lu et al. 2019 USA [61]	Multicenter longitudinal cohort study (MACS) of1727 HIV+ and HIV− patients MSM who had completed at least one study visit.	Laboratory testing and inflammatory biomarkers	CES-D: score > 20 to assess the frequency of self-reported depressive symptoms experienced over the past week.	Three EFA EIPs characterized by the following markers of immune activation: EIP-1 (sTNF-R2, sIL-2Rα, sCD27, B-cell activating factor, IP-10, sIL-6R, sCD14, and sGP130); EIP-2 (IL-6, IL-8, TNF-α, and MIP-1β); EIP-3 (MCP-1, Eotaxin, and MCP-4). In addition, BLC/BCA-1, IL-10, thymus, and activation-regulated chemokines	Biomarker levels were generally higher among HIV+ individuals; EIP-1scores were significantly associated with 9% higher odds of depressive symptoms in the HIV+ participants and 33% higher odds in the HIV− participants. Proinflammatory markers were associated with milder depression symptoms in HIV+ vs. HIV− MSM
Saylor et al. 2019 Uganda [62]	Longitudinal observational cohort study of399 HIV+ ART-naïve patients, 53% M, mean age 35 ± 8 years	50% advanced immunosuppression (CD4 count ≤ 200 cells/μL) 50% moderate immunosuppression (CD4 count 350–500 cells/μL)	CES-D	D-dimer, IL-6	The participants with advanced immunosuppression had significantly high serum levels of IL-6. IL-6 was higher among the participants with HIV-associated neurocognitive disorders or depression and in patients who died within two years.
Rubin et al. 2020 USA [63]	Longitudinal cross-over study: pharmacologic challenge study using an HPA axis probe (low-dose hydrocortisone, 10 mg) vs. placebo. 65 HIV+ patients: 36 F and 29 M, aged 18–45 years.	Not specified	DSM-IV interviews (SCID-I to determine remitted MDD status), CES-D, PCL-C, PSS-10, PSQI, and CTQ Cognitive measures (learning, memory, attention/concentration, and executive and visuospatial ability) assessed 30 min and 4 h after the placebo intervention.	Basal afternoon salivary cortisol and diurnal cortisol variations (12–6 pm). Salivary cytokines (IL-6, IL-8, IL-1β), TNF-α, CRP, IP-10, MCP-1, MIG, MMP-9, and MMP-1 concentrations	Basal cortisol concentrations higher in the remitted MDD vs. no-MDD HIV+ women and related to poorer learning and memory. Basal inflammatory cytokines [IL-6, IL-8, IL-1β, TNF-α, CRP, IFN-γ -induced protein (IP-10), monocyte chemotactic protein (MCP)-1, monokine induced by interferon (MIG), and matrix metalloproteinase (MMP)-9 and -1] higher in the remitted MDD vs. no-MDD groups of HIV+ men and women, but negatively related to cognition, independently of the remitted MDD status. Cortisol and cytokines correlated to cognition in HIV+ patients, but the associations depended on sex, remitted MDD status, and their interaction.
Saloner et al. 2020 USA [64]	Cross sectional study of123 HIV+ vs. 102 HIV− patients: 114 M and 9 F with a mean age of 40.9 years (HIV+ patients); 89 M and 13 F with a mean age of 42.1 years (HIV− patients).	HIV qualitative rapid serum test and/or Western blot confirmation of at least two of the following HIV proteins: p24, gp41, and gp120. Plasma HIV RNA via reverse transcriptase-PCR quantitation. HIV serostatus diagnosed using standard clinical antibody detection. Clinical disease severity categorized according to the CDC classification system.	BDI-II Neuropsychological assessment: verbal fluency, executive function, speed of information processing, learning, recall, working memory, and motor speed.	CSF DA and HVA concentrations. Neurocognitive assessment of verbal fluency, executive function, speed of information processing, learning, recall, working memory, and motor speed. CSF Neuroinflammatory markers: sCD14, monocyte chemotactic protein (MCP-1), interferon-inducible protein (IP-10), and neopterin.	The HIV+ patients had significantly higher BDI-II scores than the HIV− participants. The HIV+ patients exhibited higher depressive symptoms than the HIV− participants only at lower concentrations of HVA (z ≤ 0.06) and DA (z ≤ 0.11). In the HIV+ patients, a lower HVA significantly correlated with higher BDI-II scores and higher neuroinflammation, including higher MCP-1 and IP-10.
Zuñiga et al. 2020 USA [65]	Cross-sectional study of 32 HIV+ patients, with a mean age of 50.8 ± 8.8 years, 60.6% M patients, different ethnicity (White 24.2%, Black 54.5%, Latino 18.2%)	Adults clinically diagnosed with HIV for longer than 6 months and evaluated with the HIV symptom index.	PHQ-9: scores of 10 or greater indicating the presence of depression.	HbA1c, fasting glucose, IL-1β, IL-6, sIL-1RII, sIL-6R, TNF-α, sTNF-RI, sTNF-RII, CRP, adiponectin, D-dimer, fibrinogen, von Willebrand factor [vWF], and vascular function biomarkers, leptin.	Age (91%), glucose (100%), HbA1c (100%), and TNF-α (97%) were the most important variables and biomarkers for predicting depression.
Memiah et al. 2021 Tanzania [66]	Cross-sectional study of 407 HIV+ patients on ART therapy, 99 M and 308 F patients, with a median age of 48.0. (43–54) and 46.0 (42–52) years, respectively.	Survey, review of medical records, anthropometry, blood pressure assessments, and biochemical assessment of biomarkers in blood samples.	MOS-HIV	CRP, IL-6, IL-18, sTNFR-I, sTNFR-II	Elevated levels of proinflammatory cytokines, including CRP and sTNFR-II, were positively associated with self- reported mental health symptoms.
Avdoshina et al. 2021 USA [67]	Longitudinal cohort study (CHARTER study) of 933 participants subgrouped as follows: Caucasian (Non-Hispanic) and African American patients, 77% M, with a mean total age of 43 years; 14.4% with a current diagnosis of MDD, 85.6% with no MDD diagnosis.	Structured interviews and laboratory assessments	BDI-II	TrkB polymorphisms rs1212171, rs1439050, rs1187352, rs1778933, rs1443445, rs3780645, rs2378672, and rs11140800 in HIV+ patients of different race and sex.	SNP associations varied by race group and sex.
Woods et al. 2021 USA [68]	Longitudinal cohort study, derived from a NIH-funded study on prospective memory in HIV disease (R01-MH073419; Woods et al. 2020). Total cohort of 152 HIV patients: 109 HIV+, 11F and 98 M, with a mean age of 56.4 years vs. 43 HIV−, 14 F and 29 M, with a mean age of 60.7 years.	Comprehensive characterization of clinical functioning. HIV serostatus confirmed with Medmira rapid tests.	POMS	Plasma levels of BDNF and TNF-α	Lower BDNF levels were associated with higher scores on the depression–dejection and confusion–bewilderment POMS subscales among the HIV patients. Lower levels of BDNF were associated with AIDS diagnoses and CD4 count but not with viremia or duration of infection. No significant correlations emerged between BDNF and any POMS variable in the HIV− group.
Drivsholm et al. 2021 Denmark [69]	Longitudinal, observational cohort study (COCOMO) of909 HIV+ patients, 777 M and 132 F, with a mean total age of 50.8 years.	HIV infection information and use of antidepressant medications from medical charts at baseline and at 2-year follow-up.	MDI questionnaire or antidepressant use at baseline and 2-year follow-up.	Plasma levels of tryptophan, kynurenine, kynurenic acid, and quinolinic acid.	Higher quinolinic-to-kynurenic acid ratio (QKA) and higher concentrations of QA in the HIV+ patients with depression than in the HIV+ patients without depression.
Derry et al. 2022 USA [70]	Single-site observational study, patients selected from the research on older adults with HIV 2.0 multisite survey study, including143 HIV+ patients aged ≥ 50 years (mean age 61.10 years), 45 F and 98 M.	HIV diagnosis from medical charts	CES-D-10 scale UCLA Loneliness Scale HIV Stigma Scale MOS-HIV (to assess the quality of life)	Serum levels of CRP, IL-6, IFN-γ, TNF-α, IL-1β	The HIV+ patients with several depressive symptoms had higher cytokine levels [IL-6, IFN-γ, TNF-α, and IL-1β] than those with fewer depressive symptoms, were more likely to report worse physical function and more cognitive complaints than those with lower cytokine levels. CRP was not significantly correlated to these outcomes.
Saloner et al. 2022 USA [71]	Longitudinal cohort study of70 HIV+ vs. 35 HIV− patients, aged ≥ 50 years, on suppressive ART.	HIV diagnosis from medical charts	BDI Lifetime MDD diagnoses, according to the DSM-IV criteria for a depressive episode at any point in one’s lifetime	Neopterin plasma levels	The HIV+ patients had higher neopterin levels and BDI-II scores, compared to the HIV− patients. Higher neopterin plasma levels correlated with lifetime MDD.
Yang et al. 2022 China [72]	Cross-sectional study of150 HIV+ patients, 146 M and 4 F, mean age 34.8 ± 14.1 years, and7 Rhesus macaques (*n* = 5 infected with SIV, *n* = 2 healthy control monkeys).	Newly diagnosed drug-free patients; assessment not specified.	HADS	Western blotting analyses of the following: NUCB1 protein expression in the CSF of the HIV+ patients and cerebral cortex lysates from the SIV-infected and healthy control monkeys; CNR1 protein expression in cerebral cortex lysates from the SIV-infected and healthy control monkeys. Immunohistochemical analysis of NUCB1 and CNR1 expression in neurons of the temporal cortex of the SIV-infected and healthy control monkeys.	Elevated NUCB1 levels in the CSF of the HIV+ patients suffering from depression. Higher expression of NUCB1 and down-regulation of CNR1protein expression in the temporal cortex neurons of the SIV-infected monkeys vs. the healthy controls.
Shortell et al. 2022 USA [73]	Cross-sectional study of79 HIV+ patients, 35 F and 44 M, mean age 34.3 ± 8.5 years.	Medical record and clinical assessment	CES-D	Oxytocin serum concentrations	High serum oxytocin levels were associated with high total scores on CES-D and not with any of the five factors identified from the exploratory factor analysis (depressed mood, positive affect, appetite, cognitive symptoms, and perceived failure).
Anderson et al., 2022 USA [28]	Cross-sectional study MACS study of 784 M patients, 493 HIV+ vs. 291 HIV− patients, with a total median age of 53 (48–59) years.	Clinical and laboratory assessment	CES-D (score ≥ 16 identified significant depressive symptoms)	Evaluated the following inflammatory biomarkers: GlycA, CRP, IL-6, CCL2, soluble CD14 (sCD14), and soluble CD163 (sCD163).	High plasma sCD163 levels were significantly associated with severe depressive symptoms in HIV+ vs. HIV− patients
Petersen et al. 2023 USA [74]	Cross-sectional study with201 participants: 84 HIV+, 117 HIV−;137 M (mean age 43.5 ± 13.7 years), 64 F (mean age 45.2 ± 16.7 years).	HIV seropositivity using ELISA and RT-PCR tests; standardized neuro-medical, clinical evaluations, and laboratory tests.	BDI-II Current and lifetime substance use disorders measured with CIDI, using DSM-IV criteria.	Two inflammatory factors: Factor 1: IL-6, CRP, D-dimer; Factor 2: IL-8, CCL2, CXCL10.	Factor 1 and BDI-II scores were significantly associated in men (with or without HIV). The elevated plasma levels of IL-6 exclusively contributed to depression in the male patients.
Mudra Rakshasa-Loots et al. 2023 UK/Netherlands [75]	Cross-sectional study with204 participants: 125 HIV+, median age 55 (51–62), 9 F and 116 M 79 HIV−, median age 57 (52–64), 6 F and 73 M.	HIV+ patients virally suppressed, on ART therapy for at least 12 months; enrolled HIV+ patients coming from the COBRA study vs. control patients without HIV	PHQ-9	Plasma and CSF biomarkers: Kyn: Trp ratio, Neopterin, NFL, sCD14, sCD163, IL-6, IP-10/CXCL10, MCP-1/CCL2, MIG/CXCL9, MIP1α/CCL3, RANTES/CCL5, TNF-α. Additional plasma biomarkers: CRP, I-FABP, sCD16. Neuroimaging biomarkers: Choline and myo-inositol in FWM and putamen.	Prevalence and severity of depressive symptoms was significantly higher among the HIV+ participants, as well as the concentrations of several plasma and CSF inflammatory biomarkers. HIV status was associated with both an increased risk for depressive symptoms and systemic CNS inflammation. Four biomarkers of inflammation (MIG and TNF-α in the plasma, MIP1-α and IL-6 in the CSF) proved to be potential mediators of the association between HIV status and depressive symptoms.
Taylor et al. 2023 USA [76]	Cross-sectional study with151 participants (84 HIV+, 67 HIV−), subgrouped as follows: (a) HIV−/MDD− (3F, 20 M; 45.7 ± 13.5 years); (b) HIV−/MDD+ (14 F, 30 M; 45.0 ± 12.6 years); (c) HIV+/MDD− (2F, 18 M; 44.5 ± 14.3 years); and (d) HIV+/MDD+ (8F, 58 M; 46.1 ± 11.3 years).	Neuromedical and laboratory assessment	CIDI; BDI-II	Shallow-shotgun metagenomic sequencing of blood microbiome bacterial DNA.	The microbial composition did not differ between the PWH and the PWoH or between the participants with MDD and those without, though the patients with HIV infection and lifetime MDD showed a set of plasma microbiome community of the inflammatory classes, such as Flavobacteria and Nitrospira. The circulating plasma microbiome may increase the risk of MDD related to dysbiosis-induced inflammation in PWH.

**ART**: antiretroviral therapy; **BDI**: Beck Depression Inventory; **BDNF**: brain-derived neurotrophic factor; **BLC/BCA-1**: B-lymphocyte chemoattractant/B-cell-attracting chemokine 1; **CCL2/3/4/5/11**: chemokine ligand 2/3/4/5/11; **CD4/CD8**: cluster of differentiation 4/cluster differentiation 8 co-receptor glycoproteins for T-cell receptor (TCR); **CD11b**: cluster of differentiation 11b; **CDC**: Centers for Disease Control and Prevention; **CHARTER**: CNS HIV Antiretroviral Therapy Effects Research study; **CES-D**: Center for Epidemiologic Studies Depression Scale; **CHB**: chronic hepatitis B infection; **CIDI**: Composite International Diagnostic Interview; **CNR1**: cannabinoid receptor 1; **CNS**: central nervous system; **COBRA**: COmorBidity in Relation to AIDS (COBRA) study; **COCOMO**: Copenhagen Comorbidity in HIV infection study; **CRP**: C-reactive protein; **CSF**: cerebrospinal fluid; **CTQ**: Childhood Trauma Questionnaire; **CXCL1, CXCL8, CXCL9, CXCL10**: inflammatory CXC chemokines; **DA**: dopamine; **DSM-IV**: Diagnostic and Statistical Manual of Mental Disorders, fourth edition; **EFA**: exploratory factor analysis; **EIPs**: EFA-identified inflammatory processes; **ELISA**: enzyme-linked immunosorbent assay; **F** = female; **FKBP4/FKBP5**: FK506gene-binding protein 4 or 5; **FWM**: Frontal White Matter; **GAPDH**: glyceraldehyde-3-phosphate dehydrogenase; **GFAP**: glial fibrillary acidic protein; **gp**: glycoprotein; **GlycA**: glycoprotein acetyls; **G-CSF**: Granulocyte colony-stimulating factor; **GM-CSF**: Granulocyte macrophage colony-stimulating factor; **GR**: glucocorticoid receptor; **GSI**: General Severity Index; **HADS**: Hospital Anxiety and Depression Scale; **HAM-D**: Hamilton Depression Rating Scale; **HAM-A**: Hamilton Anxiety Rating Scale; **HAND**: HIV-associated neurocognitive disorder; **HIV**: human immunodeficiency virus; **HbA1c**: glycated hemoglobin; **HPA**: hypothalamic–pituitary–adrenal axis; **HVA**: homovanillic acid; **Iba1**: ionized calcium-binding adapter molecule 1; **I-FABP**: intestinal fatty acid-binding protein; **IgG**: immunoglobulin G; **IL**: interleukin; **ICV**: intra cerebro ventricular; **IDO**: indoleamine 2,3-dioxygenase; **IFN-α/IFN-γ**: interferon-alpha/interferon-gamma; **IP-10**: interferon γ-induced protein 10; **IPS-1**: IFN-β promoter stimulator-1; **KATII**: kynurenine amino transferase II; **KMO**: kynurenine 3-monooxygenase; **KYNA**: kynurenic acid; **LPS**: lipopolysaccharide; **LTMDD**: lifetime diagnosis of major depressive disorder; **M** = male; **MACS**: Multicenter AIDS Cohort Study; **MCP**: monocyte chemoattractant protein; **MDD**: major depressive disorder; **MDI**: Middle Years Development Instrument; **MHC-II**: major histocompatibility complex class II; **MIG**: monokine induced by interferon; **MINI**: Neuropsychiatric Mini-International Interview; **MIP-1α/1β**: macrophage inflammatory protein-1α/1β; **MMP**: matrix metalloproteinase; **MOS-HIV**: HIV Medical Outcomes Survey; **mRNA**: messenger ribonucleic acid; **MSM**: men who have sex with men; **MWH**: men with HIV infection; **NFL**: Neurofilament light; **Nr3c1**: nuclear receptor subfamily 3 group C member 1; **NTRK2**: gene-encoding neurotrophic tyrosine kinase receptor type 2; **NUCB1**: nucleobindin 1; **PAOFI**: Patient’s Assessment of Own Functioning Inventory; **PBMCs**: peripheral blood mononuclear cells; **PCL-C**: PTSD Checklist—Civilian Version; **PCR, RT-PCR**: polymerase chain reaction or real-time PCR; **PFC**: prefrontal cortex; **PFS-R**: Piper Fatigue Scale—Revised; **PHQ-9**: Patient Health Questionnaire-9; **POMS**: profile of mood states; **PSQI**: Pittsburgh Sleep Quality Index; **PST**: Positive Symptom Total Index; **PSDI**: Positive Symptom Distress Index; **PSS-10**: Perceived Stress Scale-10; **PWH**: people with HIV infection; **PWoH**: people without HIV infection; **QA**: quinolinic acid; **QKA**: quinolinic-to-kynurenic acid; **RIP1**: receptor-interacting protein 1; **SCID-I**: Structured Clinical Interview for DSM-IV; **SCL22A2**: Solute Carrier Family 22 Member 2; **SCL90-R**: Self-report Symptom Checklist; **sCD27, sCD14**: soluble cluster of differentiation; **sGP130**: soluble glycoProtein 130; **sIL-2Rα**: soluble interleukin-2 receptor α; **sIL-6R**: soluble interleukin-6 receptor; **SIV**: Simian Immunodeficiency Virus; **SNP**: single nucleotide polymorphism; **SPT**: sucrose preference test; **sTNF-R**: soluble tumor necrosis factor receptor; **TNF**: tumor necrosis factor; **TNF**- α: tumor necrosis factor-alpha; **Tat-HIV**: transactivator of transcription of HIV; **TrkB**: tyrosine kinase receptor B; **UCLA**: University of California, Los Angeles; **WIHS**: Women’s Interagency HIV Study; **WWH**: women with HIV infection.

**Table 2:** Clinical studies on shared biological mechanisms in patients with CHB and depression/anxiety symptoms/syndromes.

Authors, Year of Publication, Country of Study	Type of Study, Target Population	Infectious Assessment	Psychiatric Assessment	Biological Indices	Results
He et al. 2014. China [77]	Cross-sectional study of80 CHB patients with positivity for HBsAg for more than 6 months:18 F and 62 M, with median age from 26.7 to 30.8 years.	Clinical, biochemical, and virological.	PSS-10 STAI Patients subgrouped as high or low perceived stress and assessed for state and trait anxiety.	Lymphocytes count, plasma levels of ALT, IFN-*γ*, IL-10, and virus load.	Increased plasma levels of IL-10 and decreased plasma levels of IFN-γ.
Bahramabadi et al. 2017 Iran [78]	Cross-sectional study of60 patients with CHB vs. 60 healthy controls, patients’ gender not reported.	CHB diagnosed by an infectious disease specialist via previous clinical and experimental reports.	BDI-II HAM-A	Serum IL-6, IL-8, TNF- α, TGF-β levels.	Increased serum levels of IL-8, decreased serum levels of IL-6 and TGF-β in the patients with CHB and mild depression vs. the healthy controls and vs. the CHB patients without depression.
Safari-Arababadi et al. 2021. Iran [79]	Cross-sectional study of60 chronic HBV+ patients, 18–55 years old, proportion of M and F patients not specified.	Patients with HBsAg positivity for 6 months or longer; clinical assessment.	BDI-II HAM-D 17 items	IPS-1 and RIP1 mRNA levels.	IPS-1 significantly decreased in the male CHB patients with mild, moderate, or severe depression compared to the patients with no depression.

**ALT**: alanine aminotransferase; **BDI-II**: Beck Depression Inventory II; **CHB**: chronic hepatitis B; **F** = female; **HAM-A**: Hamilton Anxiety Rating Scale; **HAM-D**: Hamilton Depression Rating Scale; **HBsAg**: hepatitis B virus surface antigen; **IFN**-γ: interferon gamma; **IL**: interleukin; **IPS-1**: IFN-β promoter stimulator-1; **M** = male; **mRNA**: messenger ribonucleic acid; **NK**: natural killer cells; **PSS-10**: Perceived Stress Scale-10; **RIP1**: receptor-interacting protein 1; **STAI**: State–Trait Anxiety Inventory; **TGF-β**: transforming growth factor-beta; **TNF-α**: tumor necrosis factor-alpha.

## Data Availability

Data supporting the findings of this study are available in Table 1 and Table 2 and in the Supplementary Materials. Furthermore, the data are available from the authors on reasonable request.

## Supplementary Materials

The following supporting information can be downloaded at https://www.mdpi.com/article/10.3390/jpm13121689/s1: Figure S1: Flow diagram showing the study selection process of the included articles; Table S1: Risk of bias assessments in non-randomized clinical studies of HIV patients; and Table S2: Risk of bias assessments in non-randomized clinical studies of HBV patients. Supplementary File S1: PRISMA checklist.
