# Supplementary material for: Bidirectional Relationship between HIV/HBV Infection and Comorbid Depression and/or Anxiety: A Systematic Review on Shared Biological Mechanisms
Source: J Pers Med. 2023 Dec 5;13(12):1689. doi: 10.3390/jpm13121689 (PMC10744606; doi:10.3390/jpm13121689)
Supplement: Supplementary file 1 [file jpm-13-01689-s001.zip › jpm-2738031-Supplementary Tables S1 and S2.pdf]

Table S1. Risk of bias assessments in non-randomized clinical studies on HIV patients

Authors (Year of publication)	Type of Study	Pre-intervention domains			At-intervention Domain	Post-intervention domains			Overall Risk of bias
		Confounding Bias	*Selection bias*	*Information Bias*	*Confounding bias*	*Selection bias*	*Information Bias*	*Reporting bias*	
**Praus et al. (1990)**	Cohort study	High	Low	Moderate	No information	Low	No information	Low	High
**Tatro et al. (2010)**	Cohort study	High	Low	Moderate	Moderate	Low	No information	Low	High
**Warriner et al. (2010)**	Cohort study	Moderate	High	Moderate	Moderate	Low	No information	Low	High
**Fumaz et al. (2012)**	Cross-sectional study	High	Low	Moderate	Low	Moderate	No information	Low	High
**Avdoshina et al. (2013)**	Cohort study	High	Low	Moderate	No information	Low	No information	Low	High
**Pounder-Tandukar et al. (2014)**	Cross-sectional study	High	Low	Moderate	High	Moderate	No information	Low	High
**Cassol et al. (2015)**	Case-control study	Moderate	Low	Moderate	High	Low	No information	Low	Moderate
**Douet et al. (2016)**	Case-control study	High	No information	Moderate	High	Low	No information	Low	High
**Misinguzi et al. (2018)**	Cross-sectional study	High	High	Moderate	Moderate	Low	No information	Low	High
**Bekhbat et al. (2018)**	Cross-sectional study	High	Low	Moderate	Low	Low	No information	Low	High
**Borghetti et al. (2019)**	Cross-sectional study	High	Moderate	Moderate	Low	No information	No information	Low	High
**Lu et al. (2019)**	Cohort study	Low	Low	Moderate	Moderate	Low	No information	Low	Moderate
**Saylor et al. (2019)**	Observational study	High	High	Moderate	Moderate	Low	No information	Low	High
**Rubin et al. (2020)**	Cross-over study	Low	Low	Moderate	Low	Low	No information	Low	Moderate
**Saloner et al. (2020)**	Cross-sectional study	Moderate	Low	Moderate	Moderate	Low	No information	Low	Moderate
**Zuñiga et al. (2020)**	Cross-sectional study	Moderate	Low	Moderate	Low	Low	No information	Low	Moderate

**Memiah et al. (2021)**	Cross-sectional study	Moderate	Moderate	Low	Low	Low	No information	Low	Moderate
**Avdoshina et al. (2021)**	Prospective, observational study	Low	Low	Moderate	Moderate	Low	No information	Low	Moderate
**Woods et al. (2021)**	Cohort study	Moderate	Moderate	Moderate	Moderate	Low	No information	Low	Moderate
**Drivsholm et al. (2021)**	Longitudinal observational study	Moderate	Low	Low	Low	Low	No information	Low	Moderate
**Derry et al. (2022)**	Single-site observational study	Moderate	Low	Moderate	Moderate	Low	No information	Low	Moderate
**Saloner et al. (2022)**	Cohort study	Low	Moderate	Moderate	Low	Low	No information	Low	Moderate
**Yang et al. (2022)**	Cross-sectional study	Low	Moderate	Moderate	Low	Low	No information	Low	Moderate
**Shortell et al. (2022)**	Cross-sectional study	Moderate	Low	Moderate	Low	Low	No information	Moderate	Moderate
**Anderson et al. (2022)**	Cross-sectional study	Moderate	Low	Low	Moderate	Low	No information	Moderate	Moderate
**Petersen et al. (2023)**	Cross-sectional study	Low	Low	Low	Moderate	Low	No information	Low	Moderate
**Rakshasa-Loots et al. (2023)**	Cross-sectional study	Low	Low	Low	Moderate	Low	No information	Moderate	Moderate
**Taylor et al. (2023)**	Cross-sectional study	Moderate	Moderate	Low	High	Moderate	No information	Moderate	High

Table S2. Risk of bias assessments in non-randomized clinical studies on HBV patients

Authors (Year of publication)	Type of Study	Pre-intervention domains			At-intervention Domain	Post-intervention domains			Overall Risk of bias
		Confounding bias	*Selection bias*	*Information bias*	*Confounding bias*	*Selection bias*	*Information bias*	*Reporting bias*	
**He et al. (2014)**	Observational study	Moderate	Moderate	Moderate	Low	Low	No information	Low	Moderate
**Bahramabadi et al. (2018)**	Cross-sectional study	High	High	Moderate	Low	Low	No information	Low	High
**Safari-Arababadi et al. (2021)**	Observational study	High	High	Moderate	High	No information	No information	Low	High
