# Supplementary figures and images for: Bidirectional Relationship between HIV/HBV Infection and Comorbid Depression and/or Anxiety: A Systematic Review on Shared Biological Mechanisms
Source: J Pers Med. 2023 Dec 5;13(12):1689. doi: 10.3390/jpm13121689 (PMC10744606; doi:10.3390/jpm13121689)

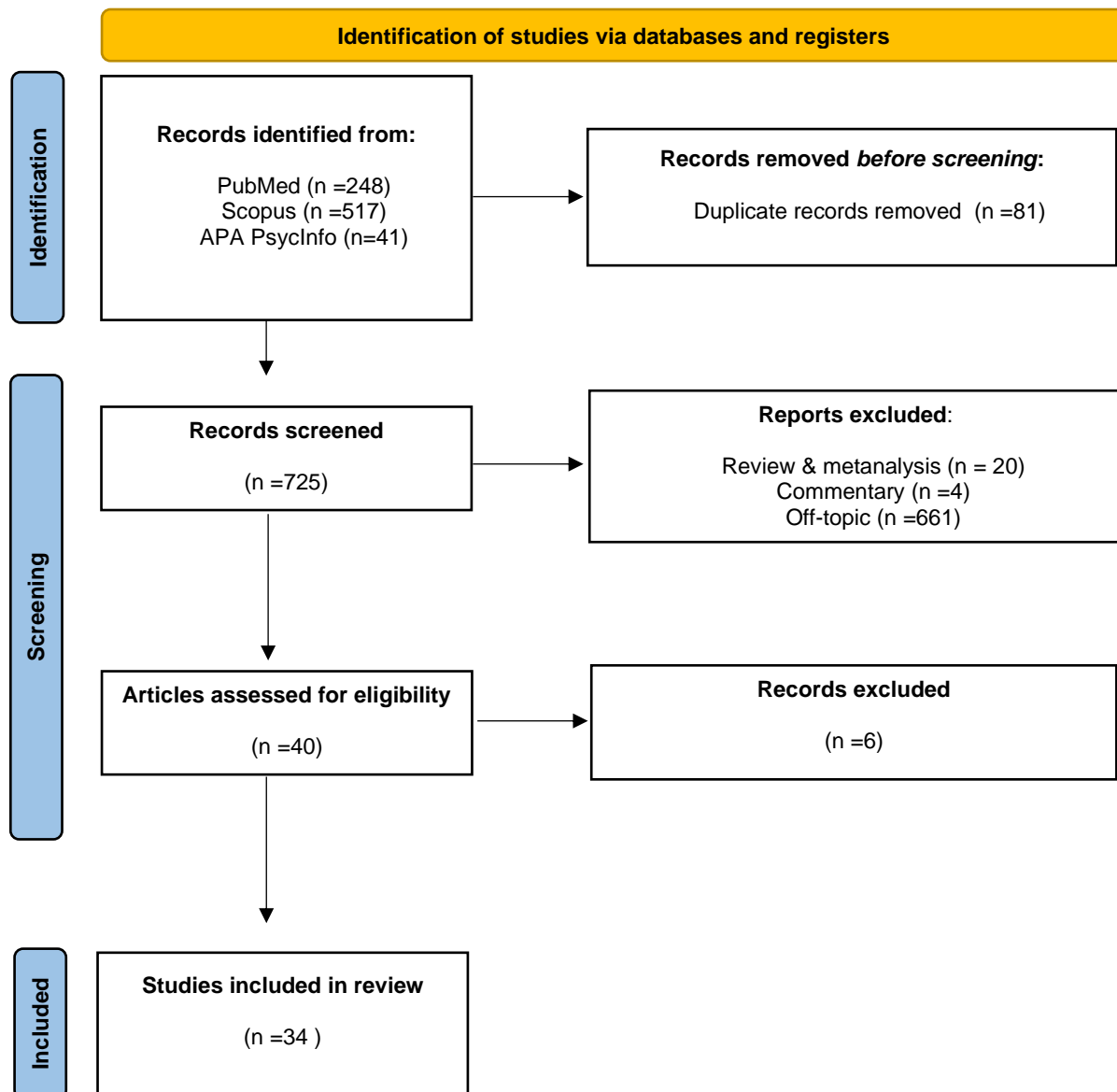

Figure S1. PRISMA flow diagram showing study selection process of included articles.

Supplement: Supplementary file 1 [file jpm-13-01689-s001.zip › jpm-2738031-Supplementary Figure S1-PRISMA_2020_flow_diagram_new_SRs_v1 (2) (1).pdf]
